# Detection of a Diverse Marine Fish Fauna Using Environmental DNA from Seawater Samples

**DOI:** 10.1371/journal.pone.0041732

**Published:** 2012-08-29

**Authors:** Philip Francis Thomsen, Jos Kielgast, Lars Lønsmann Iversen, Peter Rask Møller, Morten Rasmussen, Eske Willerslev

**Affiliations:** 1 Centre for GeoGenetics, Natural History Museum of Denmark, University of Copenhagen, Øster Voldgade, Copenhagen, Denmark; 2 Freshwater Biology Section, Department of Biology, University of Copenhagen, Helsingørgade, Hillerød, Denmark; 3 Vertebrate Department, Natural History Museum of Denmark, University of Copenhagen, Universitetsparken, Copenhagen, Denmark; University of Connecticut, United States of America

## Abstract

Marine ecosystems worldwide are under threat with many fish species and populations suffering from human over-exploitation. This is greatly impacting global biodiversity, economy and human health. Intriguingly, marine fish are largely surveyed using selective and invasive methods, which are mostly limited to commercial species, and restricted to particular areas with favourable conditions. Furthermore, misidentification of species represents a major problem. Here, we investigate the potential of using metabarcoding of environmental DNA (eDNA) obtained directly from seawater samples to account for marine fish biodiversity. This eDNA approach has recently been used successfully in freshwater environments, but never in marine settings. We isolate eDNA from ½-litre seawater samples collected in a temperate marine ecosystem in Denmark. Using next-generation DNA sequencing of PCR amplicons, we obtain eDNA from 15 different fish species, including both important consumption species, as well as species rarely or never recorded by conventional monitoring. We also detect eDNA from a rare vagrant species in the area; European pilchard (*Sardina pilchardus*). Additionally, we detect four bird species. Records in national databases confirmed the occurrence of all detected species. To investigate the efficiency of the eDNA approach, we compared its performance with 9 methods conventionally used in marine fish surveys. Promisingly, eDNA covered the fish diversity better than or equal to any of the applied conventional methods. Our study demonstrates that even small samples of seawater contain eDNA from a wide range of local fish species. Finally, in order to examine the potential dispersal of eDNA in oceans, we performed an experiment addressing eDNA degradation in seawater, which shows that even small (100-bp) eDNA fragments degrades beyond detectability within days.

Although further studies are needed to validate the eDNA approach in varying environmental conditions, our findings provide a strong proof-of-concept with great perspectives for future monitoring of marine biodiversity and resources.

## Introduction

The marine environment represents considerable value in terms of biodiversity [1] and economics through fisheries and other products derived from the sea [2], [3]. Fish are the most species-rich group of vertebrates and constitute a keystone in present-day monitoring of environmental health of marine ecosystems. Nevertheless, fish species and populations worldwide are under threat and suffer from over-exploitation [4]–[7] with considerable impact on human health [8]. Contemporary monitoring of marine fish biodiversity and resources is largely dependent on invasive and selective methods, such as bottom trawls and rotenone poisoning [9], which can only be carried out in particular areas where conditions are favourable. Furthermore, correct identification of many species across both non-commercial (e.g. Syngnathidae) and commercial (e.g. Ammodytidae) groups is problematic using traditional methods; leaving databases flawed with errors [10] and checklists incomplete [11], [12].

An alternative approach for monitoring marine fish is that of environmental DNA (eDNA), i.e. the extraction and analysis of genetic material obtained directly from environmental samples [13]. For macro-organisms, the approach was first applied to terrestrial sediment samples revealing ecosystems of extinct and extant mammals, birds, and plants [14]. Later the same approach was successfully used on ancient cave sediments [15] and ice cores [16] as well as ancient and contemporary sediments across a variety of taxa, habitats and climates [17]–[28]. Recently, eDNA from Bull frogs was successfully retrieved from contemporary pond water samples [29]. This approach has since been used to detect other amphibians [30] and invasive fish species [31] in freshwater. Furthermore, it has been demonstrated that rare and endangered freshwater insects, crustaceans, amphibians, fish and mammals can be monitored and quantified using eDNA, and that such an approach can account for entire lake faunas [32]. Despite these successful applications, the detection of macro-organisms by eDNA has to our knowledge never been reported from marine water samples.

In this study we present the first recording of marine fish biodiversity using eDNA from seawater samples.

## Results

**Figure 1:**
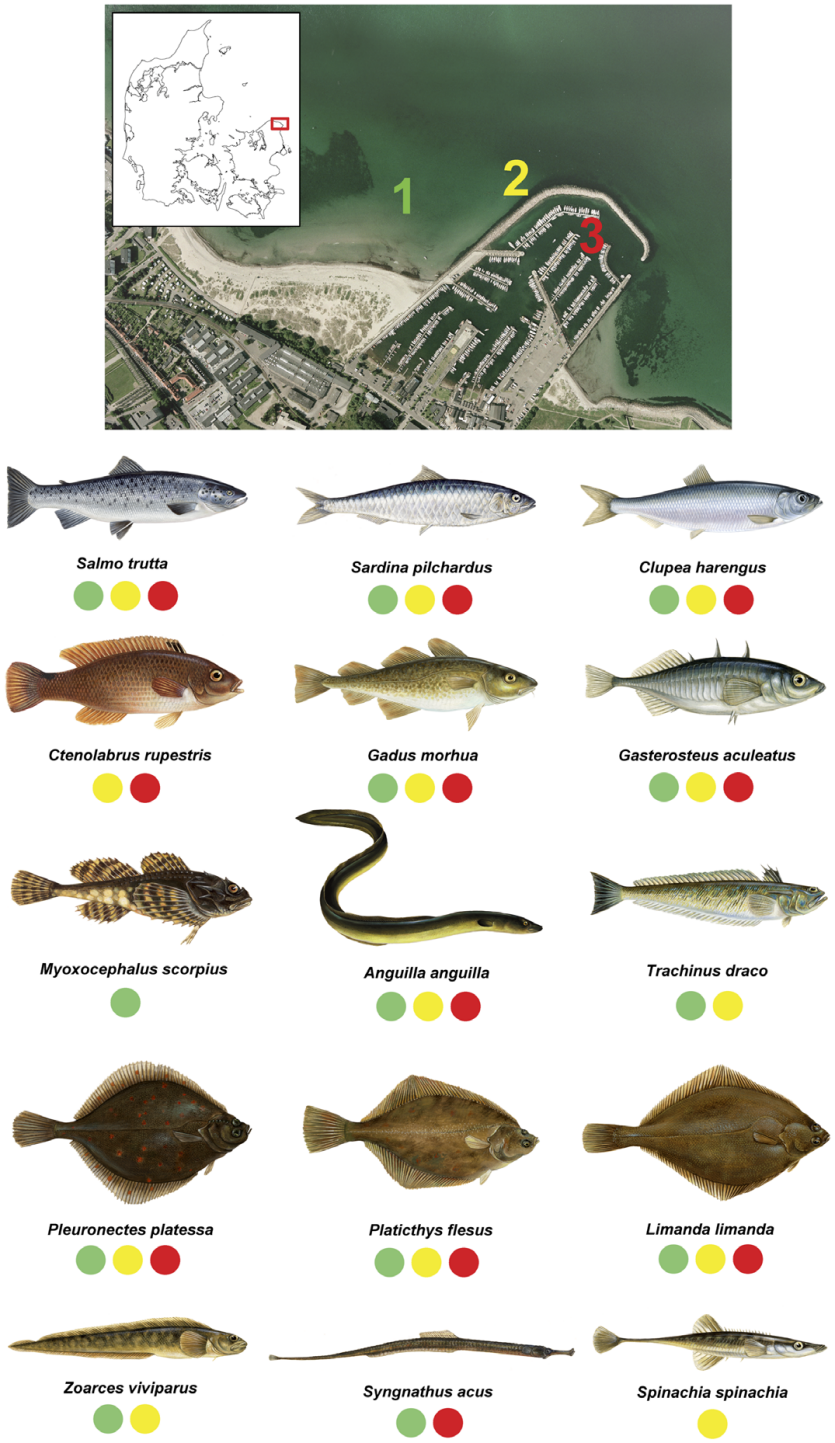
Summary of results showing sampling site and panel of fish species recovered by eDNA. Sampling locality (The Sound, Elsinore, Denmark) for this study with the three sampling sites; 1) open beach, 2) outer pier, 3) inner pier. The 15 different fish species obtained by eDNA in this study are shown with colour codes explaining in which of the three sampling sites they were found. All fish drawings by Susanne Weitemeyer ©.

Three seawater samples were collected in a temperate marine ecosystem in Denmark (Fig. 1). Samples were filtered, DNA amplified and sequenced (see Materials and Methods section). A comparison with the GenBank sequence database revealed DNA from 15 different fish species, representing a diversity of 9 orders and 11 families (Fig. 1, Table 1). These include both important consumption species, such as Atlantic cod (*Gadus morhua*), European eel (*Anguilla anguilla*), European plaice (*Pleuronectes platessa*) and Atlantic herring (*Clupea harengus*), as well as non-commercial species like Goldsinny-wrasse (*Ctenolabrus rupestris*), Shorthorn sculpin (*Myoxocephalus scorpius*) and Greater pipefish (*Syngnathus acus*). We also detected DNA from European pilchard (*Sardina pilchardus*) – a vagrant fish species in the region – and 4 species of birds, including the Red-throated loon (*Gavia stellata*), which only passes the area occasionally during migration. There was a small difference in the species composition obtained by eDNA from the three samples, with more species on the outer pier, compared to inner pier and open beach (Fig. 1).

**Table 1:** Summary of species-specific eDNA sequences recovered in this study.

Taxon	Order	Family	Species	Sequence (5′-′3)
Fish	Pleuronectiformes	Pleuronectidae	*Pleuronectes platessa*	CCGCTCGTCACGCCGCCACACATCAAGCCAGAGTGATACT
	Pleuronectiformes	Pleuronectidae	*Limanda limanda*	CCACTTGTTACACCCCCACATATCAAGCCCGAATGATATT
	Pleuronectiformes	Pleuronectidae	*Platicthys flesus*	CCACTCGTCACGCCACCACATATTAAGCCAGAGTGATACT
	Perciformes	Zoarcidae	*Zoarces viviparus*	CCACTAGTCACCCCACCCCACATCAAGCCCGAGTGGTACT
	Perciformes	Labridae	*Ctenolabrus rupestris*	TCGTACTTATGGTGGTCCCCATCCTTCACACATCTA
	Perciformes	Trachinidae	*Trachinus draco*	CCCCTAGTAACTCCTCCTCATATTAAGCCTGAATGATACT
	Anguilliformes	Anguillidae	*Anguilla anguilla*	CCAATAGTTACTCCGCCACACATTAAGCCAGAGTGGTATT
	Salmoniformes	Salmonidae	*Salmo trutta*	AACCCCCTAGTCACCCCACCTCATATCAAGCCCGAATGATACTTCCT
	Gadiformes	Gadidae	*Gadus morhua*	CCCATCGTTACCCCACCTCATGTTAAGCCCGAATGATATT
	Gasterosteiformes	Gasterosteidae	*Gasterosteus aculeatus*	CCATTAGTCACTCCACCTCACATCAAGCCTGAATGGTACT
	Gasterosteiformes	Gasterosteidae	*Spinachia spinachia*	CCATTAATTACTCCTCCTCACATTAAACCTGAATGATATT
	Syngnathiformes	Syngnathidae	*Syngnathus acus*	CCTTTAGTTACTCCTCCACATATCAAACCGGAATGATACT
	Clupeiformes	Clupeidae	*Sardina pilchardus*	CCCATGGTTACCCCACCACACATTAAGCCGGAGTGATACT
	Clupeiformes	Clupeidae	*Clupea harengus*	ATTCCGAACAAGTTGGGAGGAGTGCTTGCTCTCCTATTCTCAATT
	Scorpaeniformes	Cottidae	*Myoxocephalus scorpius*	TAGATAACGCTACACTTACCCGCTTTTTTGCC
Birds	Gaviiformes	Gaviidae	*Gavia stellata*	CCACTCGTTACACCCCCTCACATTAAGCCAGAGTGATACT
	Columbiformes	Columbidae	*Columba livia*	CCTCTAGTTACACCTCCCCATATCAAACCAGAATGATACT
	Anseriformes	Anatidae	*Cygnus olor*	AGTATCATTCTGGTTTAATGTGTGGAGGGGTTACTAGAGG
	Pelecaniformes	Phalacrocoracidae	*Phalacrocorax carbo*	CTAAAAGACATCCTAGGTTTCACACTCCTACTCCTCCTCCTAACAACAATA

All sequences are generated by pyrosequencing using Roche GS FLX 454 platform, except the 5 sequences obtained with species-specific primers (see Table 2), which are generated by cloning and subsequent Sanger sequencing. All sequences are full-length 100% match to the particular species only, identified by BLAST to the Genbank nucleotide database. Sequences are given without primers.

**Figure 2:**
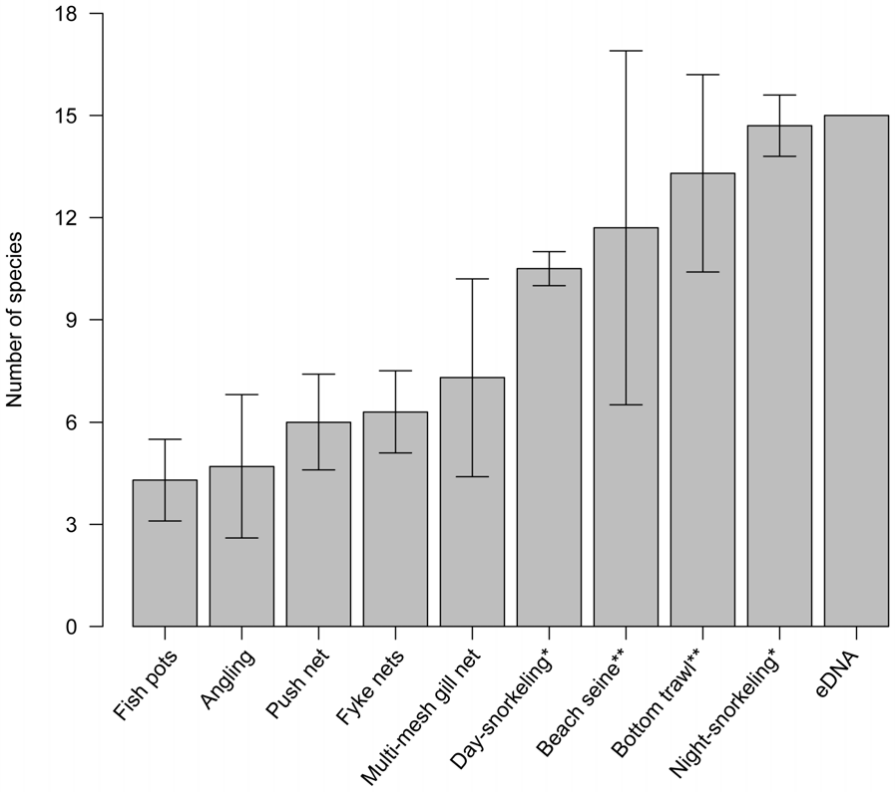
Number of fish species recorded by 9 different conventional survey methods and eDNA at The Sound of Elsinore, Denmark. Bars show mean number of fish species caught across surveys in 2009, 2010 and 2011 and error bars represent the standard deviation (see also Table S1). The eDNA bar represents the total amount of fish species recorded by this method in 2011. *) Depend heavily on competent experts in fish identification. **) Only possible where seabed conditions allow it.

As a comparison to the eDNA metabarcoding method, we conducted expert surveys in the same area using 9 different conventional methods, which yielded varying coverage of fish species diversity (Fig. 2, Table S1). Among the conventional surveillance methods, fish pots performed least efficient by uncovering on average only 4.3 fish species per sampling event, whereas night-snorkeling and bottom trawl performed the best, by detecting an average of 14.7 and 13.3 species, respectively. However, all conventional methods were outperformed or equalled by the eDNA approach finding 15 species.

**Figure 3:**
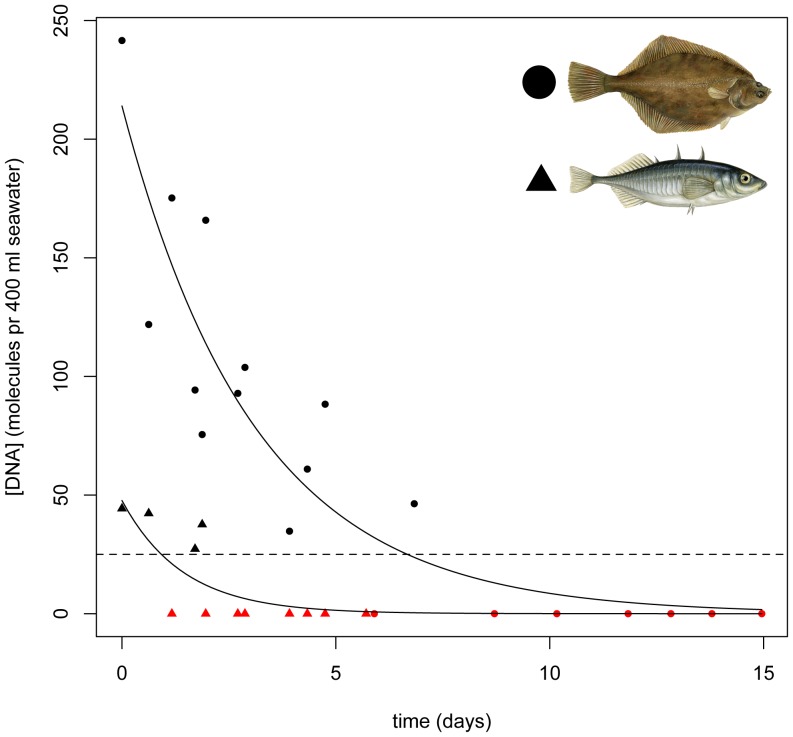
Results from eDNA degradation experiment. eDNA concentration in seawater as a function of time for the two fish species; *Platichthys flesus* (circles) and *Gasterosteus aculeatus* (triangles), investigated in a 50 l aquarium. Time points with no detection of eDNA signals are shown in red. The lines show simple exponential decay models, *p*<0.001 (*Platichthys flesus*) and *p*<0.05 (*Gasterosteus aculeatus*). Dashed line shows the suggested detection threshold of 25 DNA molecules pr 400 ml seawater. Estimated time for eDNA to degrade beyond the detection threshold was estimated to be 0.9 days for *Gasterosteus aculeatus* and 6.7 days for *Platichthys flesus*. See also Materials and Methods section.

In order to address the potential dispersal of eDNA in oceans, we performed an experiment investigating eDNA degradation. A 50 L seawater sample was collected and frequently sub-sampled for 15 days. Species-specific eDNA sequences were amplified by quantitative PCR (qPCR) for two target species (*Gasterosteus aculeatus* and *Platichthys flesus*) showing initial concentrations of 48 and 214 DNA molecules pr 400 ml seawater, respectively. Importantly, the results suggest that even very small (100-bp) eDNA fragments degrade beyond detectability within few (0.9–6.7) days (Fig. 3), (See also Material and Method section). The detection threshold below which DNA could no longer be detected was near equivalent for both species (approximately 25 DNA molecules pr 400 ml water), indicating that this may be a rough general threshold for the applied method. Average concentration of DNA in the three samples used for sequencing, was also quantified for the two target taxa, yielding similar initial concentrations (446 and 215 molecules pr 400 ml seawater for *Gasterosteus aculeatus* and *Platichthys flesus*, respectively).

All DNA extraction blanks and PCR controls performed during the experiment turned out negative, leaving no indication of contamination.

## Discussion

While it has been widely demonstrated that microbial (prokaryotic and eukaryotic) biodiversity can be studied by sequencing DNA from filtered seawater samples (e.g. [33]–[36]), we show here, for the first time, that seawater contain a high density of detectable eDNA from macro-organisms, such as fish. At the same time, it has now been demonstrated that also eDNA from whales can be obtained from seawater [37]. Targeting DNA from macro-organisms in environmental water samples is not comparable to targeting microbial organisms, as the former is present only as true eDNA (cellular debris or free DNA), whereas the latter may be detected by DNA deriving from whole, living organisms present in the water samples. The fish eDNA detected in this study most likely derives from intestinal cells, sloughed skin, scales or mucus and may consists of both free DNA, cellular debris and particle bound DNA. Animal cells exposed to the environment will quickly undergo lyses, but the specific source and relative ratio of cellular bound and free DNA is mostly studied in soil and sediment samples (e.g. [38], [39], [40]), but remains unclear in aquatic environmental samples. The filter matrix size of 0.45 µm, used in this study and [30] as well as 1.5 µm filters [31] and even 3.0 µm filters [41] have been used previously to isolate eDNA from freshwater. Considering the size of a DNA molecule, it is thus quite likely that some of the detected eDNA is particle or cellular bound.

Despite recent successful applications of eDNA detection in freshwater systems [29]–[32], [41] we find it surprising how well the approach performs on marine water samples considering: i) the larger water-volume to biomass ratio of marine ecosystems compared to that of freshwater, ii) the effects of sea-currents and wave action, and iii) the impact of salinity on the preservation and extraction of eDNA. These factors likely mean that eDNA in marine water is much less concentrated, more quickly dispersed, and may be less efficiently extracted from the water column. Still, our data reveals that marine water samples of just ½ litres yield eDNA from a variety of fish taxa, ranging from highly abundant species, such as the European plaice, to the rarely recorded vagrant species; European pilchard (Fig. 1). We found a small difference in the fish species compositions recovered by eDNA from the three different sites sampled at a very localised scale (Fig. 1). However, it remains unclear whether these differences were due to stochasticity in PCR amplification, insufficient depth of sequencing or a truly patchy occurrence of fish assemblages and their eDNA in the environment.

Importantly, when comparing results obtained with eDNA to those obtained from an array of 9 different conventional methods used in fish surveys, the eDNA approach performed remarkably well (Fig. 2). It should be noted that snorkeling, trawl and seine, which represents the methods with efficiencies closest to the eDNA approach, are either heavily dependent on competent experts in fish identification on-site (snorkeling), or only possible where seabed conditions allow it (trawl and seine). Stratified randomized bottom trawl surveys represent a cornerstone for marine monitoring in the framework of the International Council for the Exploration of the Sea [3]. These surveys cannot be carried out in shallow waters, areas with rocks, reefs, kelp or other obstacles on the seabed, and are also difficult in areas with soft sediment [42], [43]. This leaves a bias in the way marine fish faunas are monitored today, excluding important areas for biodiversity and fisheries. In contrast to many conventional methods, the eDNA method can be performed in virtually any marine habitat, and require little expertise or effort in sampling. Additionally, the molecular identification is more confident and objective than visual identification of species, which is in many cases difficult even for experts. Conversely, DNA based species identification rely on knowledge of species-specific sequences compiled by taxonomic experts. However, global initiatives addressing this need have been established, and databases are rapidly growing (http://www.boldsystems.org). A specific initiative to provide DNA barcodes of all the world's fish species was launched in 2005, and has today covered more than a third of all described species [44], [45]. It is clear that this remaining gap in knowledge will for some time impair the usefulness of eDNA monitoring in faunas where all species have not yet been DNA barcoded. On the other hand, the most complex and species-rich systems are also the most challenging to monitor with conventional methods, and likely where the advantage of eDNA will represent the most significant improvement for future monitoring.

It is obvious that sea currents may move eDNA beyond the area where species actually occur, leaving the possibility for false positive records. Also, fish predators such as birds, mammals or other fish species may distribute DNA from prey items across marine localities through defecation. Importantly however, our results from the fish eDNA degradation experiment convincingly show, that even small (100-bp) eDNA fragments in seawater persists for only a few days above detection threshold of approximately 25 molecules pr 400 ml seawater at 15°C (Fig. 3).

In freshwater, the decay of eDNA beyond the threshold of detectability has been demonstrated to happen at a scale of days or weeks [32], [46]. Notably, however, DNA degradation in seawater has previously been suggested to be substantially faster with an empirical turnover rate as low as 10 hours [47], which supports our findings and indicate lower probability of long distance dispersal of eDNA in marine ecosystems. Using an approximate degradation time of eDNA beyond detectability of minimum 12 hours and maximum 1 week, and given a rough average speed of ocean currents of 1 m/sec (normal in the Sound of Elsinore, sampled in this study), we estimate that eDNA could in this case travel between ca. 40 km–600 km in the oceans before degraded beyond detectability.

However, many other factors such as water temperature, wind speed, wind direction and local changes in currents will have great impact on the potential distance that eDNA can be transported in oceans. The average initial concentration of DNA molecules pr 400 ml seawater in the three original collected samples used for sequencing, showed similar (446 vs. 48) or very similar (215 vs. 214) values as seen in the eDNA degradation experiment for *Gasterosteus aculeatus* and *Platichthys flesus*, respectively. Given the different time that the water samples were collected for the two purposes (October 2011 vs. May 2012), it is obvious that seasonal and yearly variation as well as species phonology of *G. aculeatus* could easily account for the observed difference in eDNA for this species.

Most importantly, as a consequence of continuous dilution, the probability of detecting eDNA in marine waters very likely decreases rapidly with distance to its source, making recovery of eDNA of local origin much more plausible. Therefore, we feel convinced that eDNA obtained from marine water samples should represent only local fish fauna. This may also be the reason why we do not detect any truly exotic species (i.e. species living in deeper waters, different salinity or different latitude). Apart from the European pilchard, we only recovered eDNA from species resident to the area, suggesting that either there is no eDNA from non-resident species present, or that such DNA is too dilute to be picked up with the applied sampling procedure. The recovery of eDNA from the European pilchard, which is normally regarded as a warm-temperate species, is somewhat surprising given that the species is only rarely sighted in the sampling area. However, this species is getting increasingly more common in the northern North Sea and adjacent waters possibly due to warmer climate [48]. Furthermore, it is a species that is easily overlooked by conventional surveys due to similarity to common resident taxa. Therefore, we find it likely that the eDNA detection of European pilchard is due to authentic occurrence of the species in the area, rather than eDNA originating far from the sampling site. The recovery of eDNA from Red-throated loon (*Gavia stellata*) was also unexpected, but this finding could be authenticated by exact records in the national bird watching database (http://www.dofbasen.dk/) showing the species to be locally present at the time of sampling. We exclude the possibility of a laboratory contamination, based on negative PCR controls, extraction blanks and since no work has ever been performed on the particular species in the settings where this study was carried out. Hence, these findings illustrate how the eDNA approach may be useful in detecting unexpected species.

Despite our promising findings, it is important to emphasize that a number of issues need to be thoroughly addressed before eDNA can be considered a reliable tool for monitoring biodiversity in marine ecosystems. In particular the dispersal of eDNA in marine water must be better understood. This includes to what extent abiotic factors, like temperature and salinity, affect results. Similarly, an understanding of the phenology, changing metabolism and DNA excretion of target species may well have implications for the use of eDNA in monitoring.

It also remains untested whether the amount of eDNA molecules in marine water reflects population sizes and/or biomass of the local fauna as seen is freshwater [32], [41]. This has large applications for monitoring of marine biodiversity and in particular fisheries, where data beyond species presence is essential.

Another potential limitation for the eDNA approach is PCR primer design. It is inherent to the use of generic primers that there is a trade-off between targeting higher taxonomic levels and detecting rare sequences. Primer affinity bias leads to certain sequences (species) amplifying less efficiently than others, potentially limiting the monitoring results to species, which are expected to be locally present and are therefore used in primer design, or in general simply to species-specific sequences with the best primer affinity. However, this limitation will continuously become less crucial due to optimization and publication of primers for eDNA studies, as well as significant increase in sequencing depth and rapid advances in sequencing technology, some of which are independent of initial PCR amplification.

Regardless of many potential present limitations and a need for more basic knowledge, the eDNA approach in marine environments have widespread perspectives in terms of biodiversity monitoring and fisheries. This study provides the first evidence that a very simple eDNA based survey may offer a coverage of local marine fish faunas, which is comparatively better than, or at least as good as, any single conventional method used here. Importantly, we also demonstrate experimentally that eDNA degrades rapidly in seawater, indicating that detectable DNA is most likely of local origin. We believe that eDNA based surveys may in the future fill an important gap in broad-scale monitoring of marine biodiversity and resources.

## Materials and Methods

### Sampling locality

The study was carried out at The Sound of Elsinore, Denmark (56.04387°N, 12.61309°E) (Fig. 1).

### Conventional fish surveys

Occurrence data of fish species in the study area was obtained in late August in 2009, 2010 and 2011 by experiments led by fish expert PRM (Table S1). In order to find as many species as possible, a wide range of methods were applied each year: five fish pots, two fyke-nets, one beach-seine (width 6 m) dragged for about 100 m near shore, one multi-mesh gillnet (100*1.5 m, mesh sizes 6.5–110 mm), two hours of push netting (width 68 cm, mesh size 8 mm), two hours of angling with lures, two hours of snorkeling during the day, two hours of snorkeling at night, and half an hour of bottom trawling (width 4 m, height 1,5 m, cod-end mesh size 10 mm) from R/V Ophelia.

Permission for scientific fishing was provided by the Danish Ministry for Food, Agriculture and Fishery (journal no. 2009-02530-23088).

### Water sampling

Three 1.5-litre seawater samples were collected on October 1^st^ 2011. Samples were collected along the inner pier, along the outer pier and on open beach (Fig. 1). Samples were collected from surface water at depths of 1.5–6 m. Each sample was a pool of 30 sub-samples of each 50 ml collected along a 145 m transect, taking one sub-sample every 5 m. All samples were immediately stored at −20°C until extracted.

For the eDNA degradation experiment, a total of 50 l of seawater was collected as twenty-five 2-l samples May 16^th^ 2012 in The Sound of Elsinore (outer pier and open beach), where the original samples, used for sequencing, were also collected. The samples were pooled into a 54 l aquarium and an initial sub-sample of 400 ml was taken within one hour after sampling (t = 0). The aquarium was set up to mimic natural conditions, kept at a constant 15°C, with a 12-hour daylight cycle (standard household 15 watt neon tube) and equipped with a circulation pump powerhead (600 l/hour) ensuring full admixture and oxygenation. Subsamples of 400 ml water were taken from the aquarium at close intervals (hours – days) from May 16^th^ to May 31^st^ 2012, and all samples were immediately stored at −20°C until DNA extraction.

### DNA extraction

½ litre of each of the three 1.5-litre seawater samples was vacuum-filtered onto 47 mm diameter 0.45-µm pore size nylon filters (Osmonics, Penang, Malaysia). Immediately after, DNA was extracted from the filters using bead beating and Qiagen DNeasy Blood & Tissue Kit (using spin-column protocol). Filters were rolled up, cut into ca. 1 mm slices and placed in 2 ml tubes. 0.3 g of 0.5 mm Zirconia/Silica Beads (Biospec Products, Bartlesville, USA) and 720 µl ATL Buffer were added to each tube, which were then shaken in a Bead Beater 8 (Biospec Products, Bartlesville, USA) with 2800 oscillations/min for 45 sec. After this the tubes were incubated at 56°C for 30 min, followed by another beating and incubation step as above. Then 80 µl of Proteinase K were added to each tube followed by a final incubation step at 56°C for 2 hours with agitation. Samples were then vortexed for 15 sec and spun for 1 min (6000 g). Each supernatant (600 µl) was transferred into new 2 ml tubes. Hereafter the Qiagen DNeasy Blood & Tissue Kit (manufactures protocol) was followed for the remaining part of the DNA extraction, with the following minor adjustments; 600 µl AL Buffer, 600 µl Ethanol, and final elution steps of 2×50 µl AE Buffer for each sample.

Extraction of seawater samples for the eDNA degradation experiment was performed as above on a total of nineteen 400 ml water samples.

### PCR amplification

For PCR, two generic and four species-specific primer sets were developed to target small (<100 bp) fragments of the mitochondrial gene cytochrome b (*cytb*) in fish (Table 2). Part of the *cytb* gene was used, since GenBank had the best coverage of the local fish fauna for this genetic region. This gene has been used successfully for a similar approach in previous studies [32]. The four species-specific primers were applied since PCR using generic primers on DNA extracted from fresh tissue, showed less efficient amplification on these particular species, which are known to occur in the area. 25 µl PCR reactions were performed using 2 µl DNA extract, 10 µl TaqMan® Environmental Master Mix 2.0 (Life Technologies), 1 µl of each primer (10 µM) and 11 µl ddH_2_O under thermal conditions: 95°C for 7 min., followed by 50 cycles of 94°C for 30 sec., 50–60°C for 30 sec. and 72°C for 20 sec. completed with a final 72°C for 5 min. PCR products were verified on 2% agarose gels stained with GelRed™, and purified using a Qiagen MinElute PCR purification kit or using e-gel sizeselect 2% (Invitrogen, Life technologies, Denmark). Throughout the study we used separate laboratories for pre- and post-PCR procedures, and employed rigorous controls to monitor contamination including DNA extraction blanks and PCR blanks.

**Table 2:** Primers and probe details showing sequences, target taxa and fragment sizes.

Name	Sequence (5′-′3)	Target taxon	Fragment
Fish2bCBR	GATGGCGTAGGCAAACAAGA	Fish	80
Fish2CBL	ACAACTTCACCCCTGCAAAC		
Fish2degCBL	ACAACTTCACCCCTGCRAAY	Fish	80
Fish2CBR	GATGGCGTAGGCAAATAGGA		
ClupeaCBL	CATACGCCATTCTTCGATCA	*Clupea harengus*	85
ClupeaCBR	GGAACAAGCAGAAGGACCAG		
MyoxoCBL	GATCTGAGGCGGTTTCTCAG	*Myoxocephalus scorpius*	72
MyoxoCBR	AAGGGGAAAAGGAAGTGGAA		
SalmoCBL	CGGACAATTTTACGCCTGCC	*Salmo trutta*	87
SalmoCBR	GAAGGATTGCGTAGGCGAAT		
LabrusCBL	CGCCCTCCTATCCTCTATCC	*Ctenolabrus rupestris*	76
LabrusCBR	GAAGGTGATGCTCCGTTGTT		
TrachuCBL	CGTTCCACCCATACTTCTCC	*Phalacrocorax carbo*	92
TrachuCBR	AAGGTTTGGGGAAAATAGTGC		
GaacCBL	ACGCCACCTTAACACGTTTC	*Gasterosteus aculeatus*	101
GaacCBR	AGAGCCTGTCTGGTGAAGGA		
Gaac.probe	CTGGTGCCACACTTGTTCAC		
PlflCBL	CCGCAACAGTGATTCACCTA	*Platichthys flesus*	104
PlflCBR	TGTGAAGTAGGGGTGGAAGG		
PlflCB.probe	CCACGAAACGGGCTCAAACA		

Fragment sizes are given in base pairs including primers. All primers were designed for this study and amplify part of the Cytochrome b (*cyt-b*) gene. All regular PCRs were performed at 50°C annealing temperature and all qPCRs at 60°C annealing temperature. Probes are Minor Groove Binding (MGB) probes and have the modifications; 5′: 6-Fam (D-L-Probe), 3′: BHQ-1.

### 454 pyrosequencing

A total of six samples, each representing a pool of 8 PCR replicates with one of the two generic primer sets performed on DNA extracts from each of the three samples, were sequenced using Roche GS FLX 454 pyrosequencing. Library builds on the six samples were carried out using custom Y-shaped adaptors with MID barcode identifiers, and all reactions were performed according to protocol using NEBnext DNA Sample Prep Master Mix Set 2 (New England Biolabs, Ipswich, MA). Sequencing was carried out in accordance with manufacturer's guidelines. A total of 20,315 sequences were generated on one-half of an XLR70 PTP (Roche, Basel, Switzerland). Sequence files were sorted into separate files, by MID and primer pair, allowing 0 mismatches in the MID and up to 2 in each primer.

Sequences from pyrosequencing are uploaded to NCBI SRA: ERP001563.

### Cloning and Sanger sequencing

PCR products from amplifications using species-specific primers (see Table 2) were purified as above, cloned using Topo TA cloning kit (Invitrogen), and commercially sequenced (Macrogen, Europe).

### Sequence Identification

Extracted sequences (trimmed for primers) were compared with GenBank Nucleotide database using BLAST [49]. Taxon identification was made using MEGAN 4 [50], with following LCA settings: Min. Support = 2, Min. Score = 50, Top Percent = 2.

Only sequences with full-length 100% match to a single species were considered.

### Quantitative PCR (qPCR)

For the eDNA degradation experiment, TaqMan qPCRs were performed on a Stratagene Mx3000P.

Two species-specific sets of primers and TaqMan minor groove binding (MGB) probes were developed to target small (101–104 bp) fragments of the mitochondrial gene cytochrome b (*cytb*) in *Platichthys flesus* and *Gasterosteus aculeatus*, respectively (Table 2). The *cytb* gene was used again for the reasons given above. 25 µl qPCR reactions were performed using 2 µl DNA extract, 10 µl TaqMan® Environmental Master Mix 2.0 (Life Technologies), 1 µl of each primer (10 µM), 1 µl probe (2.5 µM) and 10 µl ddH_2_O under thermal cycling: 50°C for 5 min and 95°C for 10 min, followed by 55 cycles of 95°C for 30 sec and 60°C for 1 min.

The primer/probe systems were both validated, and tested negative on a total of 20 common saltwater fish species occurring in the area of sampling, and tested positive on the respective target species. To enable a clear quantitative interpretation of eDNA degradation, we applied species-specific TaqMan systems, which, unlike generic primers, ensures that only eDNA of the two selected target taxa were amplified. qPCR standards were prepared as a dilution series (10^−6^–10^−10^) of purified PCR products on tissue derived DNA with concentration measured on a Qubit fluorometer (Invitrogen).

Each sample was replicated in 8 independent qPCR reactions, and all positive amplifications were used in the estimation of DNA concentrations. Final concentrations in DNA molecules pr 400 ml seawater sample were calculated from the standards, setting the molecular weight of DNA to 660 g/mol/base-pair.

Efficiency of all qPCR standard curves was 90–100%.

### eDNA decay model for seawater

An exponential decay model was fitted to the qPCR data, as this is the relationship one would expect for molecular decay also used previously for similar purposes [51].

The model is the following:
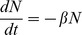
Solving this gives:
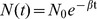
N(t) is the DNA concentration at time = t days.

The two parameters N_0_ (initial DNA concentration at time t = 0) and β (decay constant) were estimated by the nls function in R, resulting in the values N_0_ = 214 and β = 0.322, for *Platichthys flesus* and N_0_ = 48 and β = 0.701 for *Gasterosteus aculeatus*. We find a highly significant (*p*<0.001) or significant (*p*<0.05) fit to the decay models for *Platichthys flesus* and *Gasterosteus aculeatus*, respectively. Using the parameters to calculate t for N(t) = 25 (i.e. the empirically observed detection threshold), suggests that eDNA will degrade to sub-detectable levels after approximately 6.7 days for *Platichthys flesus* and 0.9 days for *Gasterosteus aculeatus*, in case of the observed initial DNA concentrations.

All statistics were performed in R ver. 2.13.1.

## Supporting Information

Table S1 **Species list and details for conventional fish surveys.**
(PDF)
