# Supplementary material for: Detection of a Diverse Marine Fish Fauna Using Environmental DNA from Seawater Samples
Source: PLoS One. 2012 Aug 29;7(8):e41732. doi: 10.1371/journal.pone.0041732 (PMC3430657; doi:10.1371/journal.pone.0041732)
Supplement: Table S1 — Species list and details for conventional fish surveys. (PDF) [file pone.0041732.s001.pdf]

Supporting Information, Table S1:

Detection of a Diverse Marine Fish Fauna using Environmental DNA from Seawater Samples

Philip Francis Thomsen[1]* Jos Kielgast[1], Lars Lønsmann Iversen[2], Peter Rask Møller[3], Morten Rasmussen[1], Eske Willerslev[1]*

[1] Centre for GeoGenetics, Natural History Museum of Denmark, University of Copenhagen, Øster Voldgade 5-7, DK-1350 Copenhagen, Denmark.

[2] Freshwater Biology Section, Department of Biology, University of Copenhagen, Helsingørgade 51, DK-3400 Hillerød, Denmark.

[3] Vertebrate Department, Natural History Museum of Denmark, University of Copenhagen, Universitetsparken 15, DK-2100 Copenhagen, Denmark.

E-mail: ewillerslev@snm.ku.dk (EW); pfthomsen@snm.ku.dk (PFT)

Table S1. Results of fish surveys based on conventional methods at The Sound of Elsinore, Denmark 2009-2011. Species caught are listed by survey method for each year along with exact date, coordinates of sampling and approximate depth.

Year	Species	Survey method	Depth	Date	Latitude	Longitude
2009	*Belone belone*	Angling	18.3	17.08.09	55.97722	12.62916
2009	*Clupea harengus*	Angling	20	17.08.09	55.97722	12.62916
2009	*Ammodytes tobianus*	Beach seine	2	18.08.09	56.04177	12.61857
2009	*Clupea harengus*	Beach seine	2	18.08.09	56.04177	12.61857
2009	*Platichthys flesus*	Beach seine	2	18.08.09	56.04177	12.61857
2009	*Pleuronectes platessa*	Beach seine	2	18.08.10	56.04177	12.61857
2009	*Pomatoschistus minutus*	Beach seine	2	18.08.09	56.04177	12.61857
2009	*Scopthalmus rhombus*	Beach seine	2	18.08.09	56.04177	12.61857
2009	*Spinachia spinachia*	Beach seine	2	18.08.09	56.04177	12.61857
2009	*Sprattus sprattus*	Beach seine	2	18.08.09	56.04177	12.61857
2009	*Syngnathus rostellatus*	Beach seine	2	18.08.09	56.04177	12.61857
2009	*Syngnathus typhle*	Beach seine	2	18.08.09	56.04177	12.61857
2009	*Anguilla anguilla*	Fish pot	3	18.08.09	56.04177	12.61857
2009	*Cobius niger*	Fish pot	3	18.08.09	56.04177	12.61857
2009	*Ctenolabrus rupestris*	Fish pot	3	18.08.09	56.04177	12.61857
2009	*Pholis gunellus*	Fish pot	3	18.08.09	56.04177	12.61857
2009	*Taurulus bubalis*	Fish pot	3	18.08.09	56.04177	12.61857
2009	*Zoarces viviparus*	Fish pot	3	18.08.09	56.04177	12.61857
2009	*Ciliata mustela*	Fyke	2	18.08.09	56.04177	12.61857
2009	*Gadus morhua*	Fyke	2	18.08.09	56.04177	12.61857
2009	*Scopthalmus rhombus*	Fyke	2	18.08.09	56.04177	12.61857
2009	*Symphodus melops*	Fyke	2	18.08.09	56.04177	12.61857
2009	*Zoarces viviparus*	Fyke	2	18.08.09	56.04177	12.61857
2009	*Ammodytes marinus*	Multi-mesh gill net	3	18.08.09	56.04177	12.61857
2009	*Ammodytes tobianus*	Multi-mesh gill net	2	17.08.09	56.04177	12.61857
2009	*Belone belone*	Multi-mesh gill net	3	18.08.09	56.04177	12.61857
2009	*Clupea harengus*	Multi-mesh gill net	3	18.08.09	56.04177	12.61857
2009	*Gasterosteus aculeatus*	Multi-mesh gill net	3	18.08.09	56.04177	12.61857
2009	*Hyperoplus lanceolatus*	Multi-mesh gill net	3	18.08.09	56.04177	12.61857
2009	*Trachinus draco*	Multi-mesh gill net	3	18.08.09	56.04177	12.61857
2009	*Platichthys flesus*	Push net	1	17.08.09	56.04177	12.61857
2009	*Scopthalmus rhombus*	Push net	1	17.08.09	56.04177	12.61857
2009	*Solea solea*	Push net	1	17.08.09	56.04177	12.61857
2009	*Spinachia spinachia*	Push net	1	17.08.09	56.04177	12.61857
2009	*Syngnathus rostellatus*	Push net	1	17.08.09	56.04177	12.61857
2009	*Ammodytes tobianus*	Snorkling day	1-3	17.08.09	56.04177	12.61857
2009	*Ctenolabrus rupestris*	Snorkling day	1-3	17.08.09	56.04177	12.61857

2009	*Gasterosteus aculeatus*	Snorkling day	1-3	17.08.09	56.04177	12.61857
2009	*Platichthys flesus*	Snorkling day	1-3	17.08.09	56.04177	12.61857
2009	*Pomatoschistus minutus*	Snorkling day	1-3	17.08.09	56.04177	12.61857
2009	*Psetta maxima*	Snorkling day	1-3	17.08.09	56.04177	12.61857
2009	*Spinachia spinachia*	Snorkling day	1-3	17.08.09	56.04177	12.61857
2009	*Symphodus melops*	Snorkling day	1-3	17.08.09	56.04177	12.61857
2009	*Syngnathus rostellatus*	Snorkling day	1-3	17.08.09	56.04177	12.61857
2009	*Anguilla anguilla*	Snorkling night	0-5	18.08.09	56.01055	12.59442
2009	*Aphia minuta*	Snorkling night	0-5	18.08.09	56.01055	12.59442
2009	*Cobius niger*	Snorkling night	0-5	18.08.09	56.01055	12.59442
2009	*Gadus morhua*	Snorkling night	0-5	18.08.09	56.01055	12.59442
2009	*Gobiusculus flavescens*	Snorkling night	0-5	18.08.09	56.01055	12.59442
2009	*Limanda limanda*	Snorkling night	0-5	18.08.09	56.01055	12.59442
2009	*Platichthys flesus*	Snorkling night	0-5	18.08.09	56.01055	12.59442
2009	*Pomatoschistus minutus*	Snorkling night	0-5	18.08.09	56.01055	12.59442
2009	*Salmo trutta*	Snorkling night	0-5	18.08.09	56.01055	12.59442
2009	*Spinachia spinachia*	Snorkling night	0-5	18.08.09	56.01055	12.59442
2009	*Syngnathus acus*	Snorkling night	0-5	18.08.09	56.01055	12.59442
2009	*Syngnathus typhle*	Snorkling night	0-5	18.08.09	56.01055	12.59442
2009	*Taurulus bubalis*	Snorkling night	0-5	18.08.09	56.01055	12.59442
2009	*Zoarces viviparus*	Snorkling night	0-5	18.08.09	56.01055	12.59442
2009	*Clupea harengus*	trawl	18.3	17.08.09	55.97722	12.62916
2009	*Eutrigla gurnardus*	trawl	18.3	17.08.09	55.97722	12.62916
2009	*Gadus morhua*	trawl	18.3	17.08.09	55.97722	12.62916
2009	*Hippoglossoides platessoides*	trawl	18.3	17.08.09	55.97722	12.62916
2009	*Limanda limanda*	trawl	18.3	17.08.09	55.97722	12.62916
2009	*Melanogrammus aeglefinus*	trawl	18.3	17.08.09	55.97722	12.62916
2009	*Merlangius merlangus*	trawl	18.3	17.08.09	55.97722	12.62916
2009	*Microstomus kitt*	trawl	18.3	17.08.09	55.97722	12.62916
2009	*Platichthys flesus*	trawl	18.3	17.08.09	55.97722	12.62916
2009	*Pleuronectes platessa*	trawl	18.3	17.08.09	55.97722	12.62916
2009	*Solea solea*	trawl	18.3	17.08.09	55.97722	12.62916
2010	*Belone belone*	Angling	31	23.08.10	55.97722	12.62916
2010	*Clupea harengus*	Angling	31	23.08.10	55.97722	12.62916
2010	*Gadus morhua*	Angling	31	23.08.10	55.97722	12.62916
2010	*Hyperoplus lanceolatus*	Angling	5-9	24.08.10	56.09243	12.50063
2010	*Pollachius pollachius*	Angling	30	24.08.10	55.97722	12.62916
2010	*Scomber scombrus*	Angling	31	23.08.10	55.97722	12.62916
2010	*Trachinus draco*	Angling	5-9	24.08.10	56.09243	12.50063
2010	*Ammodytes tobianus*	Beach seine	0-2	24.08.10	56.04177	12.61857
2010	*Engraulis encrasicolus*	Beach seine	0-2	24.08.10	56.04177	12.61857
2010	*Platichthys flesus*	Beach seine	0-2	24.08.10	56.04177	12.61857
2010	*Pomatoschistus minutus*	Beach seine	0-2	24.08.10	56.04177	12.61857

2010	*Spinachia spinachia*	Beach seine	0-2	24.08.10	56.04177	12.61857
2010	*Symphodus melops*	Beach seine	0-2	24.08.10	56.04177	12.61857
2010	*Syngnathus acus*	Beach seine	0-2	24.08.10	56.04177	12.61857
2010	*Cobius niger*	Fish pot	5	24.08.10	56.04177	12.61857
2010	*Ctenolabrus rupestris*	Fish pot	5	24.08.10	56.04177	12.61857
2010	*Perca fluviatilis*	Fish pot	5	24.08.10	56.04177	12.61857
2010	*Symphodus melops*	Fish pot	5	24.08.10	56.04177	12.61857
2010	*Cobius niger*	Fyke	3	24.08.10	56.04177	12.61857
2010	*Ctenolabrus rupestris*	Fyke	3	24.08.10	56.04177	12.61857
2010	*Platichthys flesus*	Fyke	3	24.08.10	56.04177	12.61857
2010	*Scopthalmus rhombus*	Fyke	3	24.08.10	56.04177	12.61857
2010	*Symphodus melops*	Fyke	3	24.08.10	56.04177	12.61857
2010	*Zoarces viviparus*	Fyke	3	24.08.10	56.04177	12.61857
2010	*Clupea harengus*	Multi-mesh gill net	3	24.08.10	56.04177	12.61857
2010	*Perca fluviatilis*	Multi-mesh gill net	3	24.08.10	56.04177	12.61857
2010	*Salmo trutta*	Multi-mesh gill net	3	24.08.10	56.04177	12.61857
2010	*Trachinus draco*	Multi-mesh gill net	3	24.08.10	56.04177	12.61857
2010	*Platichthys flesus*	Push net	1	23.08.10	56.04177	12.61857
2010	*Pleuronectes platessa*	Push net	1	23.08.10	56.04177	12.61857
2010	*Psetta maxima*	Push net	1	23.08.10	56.04177	12.61857
2010	*Scopthalmus rhombus*	Push net	1	23.08.10	56.04177	12.61857
2010	*Solea solea*	Push net	1	23.08.10	56.04177	12.61857
2010	*Anguilla anguilla*	Snorkling day	2	23.08.10	56.04177	12.61857
2010	*Cobius niger*	Snorkling day	2	23.08.10	56.04177	12.61857
2010	*Ctenolabrus rupestris*	Snorkling day	2	23.08.10	56.04177	12.61857
2010	*Gobiusculus flavescens*	Snorkling day	2	23.08.10	56.04177	12.61857
2010	*Platichthys flesus*	Snorkling day	2	23.08.10	56.04177	12.61857
2010	*Pomatoschistus minutus*	Snorkling day	2	23.08.10	56.04177	12.61857
2010	*Salmo trutta*	Snorkling day	2	23.08.10	56.04177	12.61857
2010	*Scopthalmus rhombus*	Snorkling day	2	23.08.10	56.04177	12.61857
2010	*Syngnathus acus*	Snorkling day	2	23.08.10	56.04177	12.61857
2010	*Syngnathus rostellatus*	Snorkling day	2	23.08.10	56.04177	12.61857
2010	*Ammodytes tobianus*	Snorkling night	1-7	23.08.10	56.04426	12.6135
2010	*Anguilla anguilla*	Snorkling night	1-7	23.08.10	56.04426	12.6135
2010	*Belone belone*	Snorkling night	1-7	23.08.10	56.04426	12.6135
2010	*Clupea harengus*	Snorkling night	1-7	23.08.10	56.04426	12.6135
2010	*Ctenolabrus rupestris*	Snorkling night	1-7	23.08.10	56.04426	12.6135
2010	*Gadus morhua*	Snorkling night	1-7	23.08.10	56.04426	12.6135
2010	*Myoxocephalus scorpius*	Snorkling night	1-7	23.08.10	56.04426	12.6135
2010	*Platichthys flesus*	Snorkling night	1-7	23.08.10	56.04426	12.6135
2010	*Pleuronectes platessa*	Snorkling night	1-7	23.08.10	56.04426	12.6135
2010	*Pomatoschistus minutus*	Snorkling night	1-7	23.08.10	56.04426	12.6135
2010	*Salmo trutta*	Snorkling night	1-7	23.08.10	56.04426	12.6135

2010	*Solea solea*	Snorkling night	1-7	23.08.10	56.04426	12.6135
2010	*Spinachia spinachia*	Snorkling night	1-7	23.08.10	56.04426	12.6135
2010	*Trachinus draco*	Snorkling night	1-7	23.08.10	56.04426	12.6135
2010	*Callionymus lyra*	trawl	21	23.08.10	55.97722	12.62916
2010	*Clupea harengus*	trawl	21	23.08.10	55.97722	12.62916
2010	*Eutrigla gurnardus*	trawl	21	23.08.10	55.97722	12.62916
2010	*Gadus morhua*	trawl	21	23.08.10	55.97722	12.62916
2010	*Hippoglossoides platessoides*	trawl	21	23.08.10	55.97722	12.62916
2010	*Limanda limanda*	trawl	21	23.08.10	55.97722	12.62916
2010	*Merlangius merlangus*	trawl	21	23.08.10	55.97722	12.62916
2010	*Microstomus kitt*	trawl	21	23.08.10	55.97722	12.62916
2010	*Platichthys flesus*	trawl	21	23.08.10	55.97722	12.62916
2010	*Pleuronectes platessa*	trawl	21	23.08.10	55.97722	12.62916
2010	*Pomatoschistus minutus*	trawl	21	23.08.10	55.97722	12.62916
2010	*Trachinus draco*	trawl	21	23.08.10	55.97722	12.62916
2010	*Zeugopterus punctatus*	trawl	21	23.08.10	55.97722	12.62916
2011	*Clupea harengus*	Angling	25	23.08.11	55.97722	12.62916
2011	*Gadus morhua*	Angling	21	22.08.11	56.04177	12.61857
2011	*Merlangius merlangus*	Angling	21	22.08.11	56.04177	12.61857
2011	*Scomber scombrus*	Angling	25	23.08.11	55.97722	12.62916
2011	*Trachinus draco*	Angling	21	22.08.11	56.04177	12.61857
2011	*Ammodytes tobianus*	Beach seine	0-10	22.08.11	56.04177	12.61857
2011	*Entelurus aequoreus*	Beach seine	0-10	22.08.11	56.04177	12.61857
2011	*Gadus morhua*	Beach seine	0-10	22.08.11	56.04177	12.61857
2011	*Gasterosteus aculeatus*	Beach seine	0-10	22.08.11	56.04177	12.61857
2011	*Gobiusculus flavescens*	Beach seine	0-10	22.08.11	56.04177	12.61857
2011	*Hyperoplus lanceolatus*	Beach seine	0-10	22.08.11	56.04177	12.61857
2011	*Nerophis ophidion*	Beach seine	0-10	22.08.11	56.04177	12.61857
2011	*Pholis gunellus*	Beach seine	0-10	22.08.11	56.04177	12.61857
2011	*Platichthys flesus*	Beach seine	0-10	22.08.11	56.04177	12.61857
2011	*Pleuronectes platessa*	Beach seine	0-10	22.08.11	56.04177	12.61857
2011	*Pomatoschistus minutus*	Beach seine	0-10	22.08.11	56.04177	12.61857
2011	*Psetta maxima*	Beach seine	0-10	22.08.11	56.04177	12.61857
2011	*Scopthalmus rhombus*	Beach seine	0-10	22.08.11	56.04177	12.61857
2011	*Solea solea*	Beach seine	0-10	22.08.11	56.04177	12.61857
2011	*Spinachia spinachia*	Beach seine	0-10	22.08.11	56.04177	12.61857
2011	*Syngnathus acus*	Beach seine	0-10	22.08.11	56.04177	12.61857
2011	*Syngnathus rostellatus*	Beach seine	0-10	22.08.11	56.04177	12.61857
2011	*Syngnathus typhle*	Beach seine	0-10	22.08.11	56.04177	12.61857
2011	*Zoarces viviparus*	Beach seine	0-10	22.08.11	56.04177	12.61857
2011	*Anguilla anguilla*	Fish pot	5	23.08.11	56.04177	12.61857
2011	*Ctenolabrus rupestris*	Fish pot	5	23.08.11	56.04426	12.6135
2011	*Myoxocephalus scorpius*	Fish pot	5	23.08.11	56.04426	12.6135

2011	*Anguilla anguilla*	Fyke	3	23.08.11	56.04177	12.61857
2011	*Ctenolabrus rupestris*	Fyke	3	23.08.11	56.04177	12.61857
2011	*Platichthys flesus*	Fyke	3	23.08.11	56.04177	12.61857
2011	*Scopthalmus rhombus*	Fyke	3	23.08.11	56.04177	12.61857
2011	*Solea solea*	Fyke	3	23.08.11	56.04177	12.61857
2011	*Symphodus melops*	Fyke	3	23.08.11	56.04177	12.61857
2011	*Syngnathus acus*	Fyke	3	23.08.11	56.04177	12.61857
2011	*Zoarces viviparus*	Fyke	3	23.08.11	56.04177	12.61857
2011	*Clupea harengus*	Multi-mesh gill net	2-5	23.08.11	56.04177	12.61857
2011	*Cobius niger*	Multi-mesh gill net	2-5	23.08.11	56.04177	12.61857
2011	*Ctenolabrus rupestris*	Multi-mesh gill net	2-5	23.08.11	56.04177	12.61857
2011	*Gadus morhua*	Multi-mesh gill net	2-5	23.08.11	56.04177	12.61857
2011	*Gasterosteus aculeatus*	Multi-mesh gill net	2-5	23.08.11	56.04177	12.61857
2011	*Hyperoplus lanceolatus*	Multi-mesh gill net	2-5	23.08.11	56.04177	12.61857
2011	*Merlangius merlangus*	Multi-mesh gill net	2-5	23.08.11	56.04177	12.61857
2011	*Pollachius pollachius*	Multi-mesh gill net	2-5	23.08.11	56.04177	12.61857
2011	*Pollachius virens*	Multi-mesh gill net	2-5	23.08.11	56.04177	12.61857
2011	*Spinachia spinachia*	Multi-mesh gill net	2-5	23.08.11	56.04177	12.61857
2011	*Trachinus draco*	Multi-mesh gill net	2-5	23.08.11	56.04177	12.61857
2011	*Ammodytes tobianus*	Push net	0-1	22.08.11	56.04177	12.61857
2011	*Pleuronectes platessa*	Push net	0-1	22.08.11	56.04177	12.61857
2011	*Pomatoschistus microps*	Push net	0-1	22.08.11	56.04177	12.61857
2011	*Scopthalmus rhombus*	Push net	0-1	22.08.11	56.04177	12.61857
2011	*Solea solea*	Push net	0-1	22.08.11	56.04177	12.61857
2011	*Symphodus melops*	Push net	0-1	22.08.11	56.04177	12.61857
2011	*Syngnathus rostellatus*	Push net	0-1	22.08.11	56.04177	12.61857
2011	*Zoarces viviparus*	Push net	0-1	22.08.11	56.04177	12.61857
2011	*Anguilla anguilla*	Snorkling night	1-3	22.08.11	56.01055	12.59442
2011	*Belone belone*	Snorkling night	1-3	22.08.11	56.01055	12.59442
2011	*Clupea harengus*	Snorkling night	1-3	22.08.11	56.01055	12.59442
2011	*Cobius niger*	Snorkling night	1-3	22.08.11	56.01055	12.59442
2011	*Gadus morhua*	Snorkling night	1-3	22.08.11	56.01055	12.59442
2011	*Gasterosteus aculeatus*	Snorkling night	1-3	22.08.11	56.01055	12.59442
2011	*Myoxocephalus scorpius*	Snorkling night	1-3	22.08.11	56.01055	12.59442
2011	*Platichthys flesus*	Snorkling night	1-3	22.08.11	56.01055	12.59442
2011	*Pomatoschistus minutus*	Snorkling night	1-3	22.08.11	56.01055	12.59442
2011	*Salmo trutta*	Snorkling night	1-3	22.08.11	56.01055	12.59442
2011	*Scopthalmus rhombus*	Snorkling night	1-3	22.08.11	56.01055	12.59442
2011	*Spinachia spinachia*	Snorkling night	1-3	22.08.11	56.01055	12.59442
2011	*Syngnathus rostellatus*	Snorkling night	1-3	22.08.11	56.01055	12.59442
2011	*Syngnathus typhle*	Snorkling night	1-3	22.08.11	56.01055	12.59442
2011	*Taurulus bubalis*	Snorkling night	1-3	22.08.11	56.01055	12.59442
2011	*Zoarces viviparus*	Snorkling night	1-3	22.08.11	56.01055	12.59442

2011	*Agonus cataphractus*	Trawl	12	23.08.11	56.09243	12.50063
2011	*Callionymus lyra*	Trawl	12	23.08.11	56.09243	12.50063
2011	*Cobius niger*	Trawl	21	22.08.11	55.97722	12.62916
2011	*Eutrigla gurnardus*	Trawl	21	22.08.11	55.97722	12.62916
2011	*Gadus morhua*	Trawl	21	22.08.11	55.97722	12.62916
2011	*Hippoglossoides platessoides*	Trawl	21	22.08.11	55.97722	12.62916
2011	*Limanda limanda*	Trawl	21	22.08.11	55.97722	12.62916
2011	*Liparis sp.*	Trawl	21	22.08.11	55.97722	12.62916
2011	*Melanogrammus aeglefinus*	Trawl	21	22.08.11	55.97722	12.62916
2011	*Merlangius merlangus*	Trawl	21	22.08.11	55.97722	12.62916
2011	*Microstomus kitt*	Trawl	21	22.08.11	55.97722	12.62916
2011	*Platichthys flesus*	Trawl	21	22.08.11	55.97722	12.62916
2011	*Pleuronectes platessa*	Trawl	12	23.08.11	56.09243	12.50063
2011	*Psetta maxima*	Trawl	12	23.08.11	56.09243	12.50063
2011	*Solea solea*	Trawl	21	22.08.11	55.97722	12.62916
2011	*Sprattus sprattus*	Trawl	21	22.08.11	55.97722	12.62916
2011	*Trachinus draco*	Trawl	12	23.08.11	56.09243	12.50063
